# A cost-consequence analysis of normalised advance care planning practices among people with chronic diseases in hospital and community settings

**DOI:** 10.1186/s12913-021-06749-x

**Published:** 2021-07-23

**Authors:** Sarah Jeong, Se Ok Ohr, Peter Cleasby, Tomiko Barrett, Ryan Davey, Simon Deeming

**Affiliations:** 1 School of Nursing and Midwifery, University of Newcastle, 10 Chittaway Road, Ourimbah, NSW 2258 Australia; 2 Hunter New England Nursing and Midwifery Research Centre, Hunter New England Local Health District, James Fletcher Campus, Gate Cottage, 72 Watt St, Newcastle, NSW 2300 Australia; 3 Division of Aged, Subacute and Complex Care, PO Box 6088 Long Jetty, Central Coast Local Health District, NSW 2261 Gosford, Australia; 4 Department of Aged Care Services, Wyong Hospital, PO Box 4200, Lakehaven, Central Coast Local Health District, Wyong, NSW 2263 Australia; 5 Hunter Medical Research Institute, Lot 1, Kookaburra Circuit, New Lambton Heights, Newcastle, NSW 2305 Australia

**Keywords:** Advance care directive, Advance care planning, Chronic disease, Community, Hospital, Clinical trial, Cost-consequence analysis, Nurses

## Abstract

**Background:**

A growing body of international literature concurs that comprehensive and complex Advance Care Planning (ACP) programs involving specially qualified or trained healthcare professionals are effective in increasing documentation of Advance Care Directives (ACDs), improving compliance with patients’ wishes and satisfaction with care, and quality of care for patients and their families. Economic analyses of ACDs and ACP have been more sporadic and inconclusive. This study aimed to contribute to the evidence on resource use associated with implementation of ACP and to inform key decision-makers of the resource implications through the conduct of a cost-consequence analysis of the Normalised Advance Care Planning (NACP) trial.

**Methods:**

The outcomes for the economic evaluation included the number of completed “legally binding” ACDs and the number of completed Conversation Cards (CC). The cost analysis assessed the incremental difference in resource utilisation between Usual Practice and the Intervention. Costs have been categorised into: 1) Contract staff costs; 2) Costs associated with the development of the intervention; 3) Implementation costs; 4) Intervention (delivery) costs; and 5) Research costs.

**Results:**

The cost incurred for each completed ACD was A$13,980 in the hospital setting and A$1248 in the community setting. The cost incurred for each completed Conversation Card was A$7528 in the hospital setting and A$910 in the community setting.

**Conclusions:**

The cost-consequence analysis does not support generalisation of the specified intervention within the hospital setting. The trial realised an estimated incremental cost per completed ACD of $1248, within the community setting. This estimate provides an additional benchmark against which decision-makers can assess the value of either 1) this approach towards the realisation of additional completed ACDs; and/or 2) the value of ACP and ACDs more broadly, when this estimate is positioned within the potential health outcomes and downstream health service implications that may arise for people with or without a completed ACD.

**Trial registration:**

The study was retrospectively registered with the Australian New Zealand Clinical Trials Registry (Trial ID: ACTRN12618001627246). The URL of the trial registry record.

**Supplementary Information:**

The online version contains supplementary material available at 10.1186/s12913-021-06749-x.

## Background

Nancy Cruzan who was in a persistent vegetative state for seven years after a car accident in 1983 became a figure in the right-to-die movement and provided an incentive to discussion on Advance Directives (ADs) in the United States (US) [1]. The US government promoted ADs to encourage members of the public to be proactive about expressing their end-of-life treatment wishes and a statutory basis was established in all 50 states, under the *Patient Self-Determination Act (PSDA)* in 1991 [2]. Although there are variations in the terms, for example, ADs in US [2] and Germany [3], and Advance Care Directives (ACDs) or Advance Health Directives in Australia [4], the need for and importance of end-of-life care decisions have ever since grown and have been contributed by a variety of factors. Among these are the ageing population [5–7], new life-sustaining technologies [8], rapidly increasing health-care costs [9], increasing patient awareness and demands for autonomy [10, 11], and the increasing numbers of people with chronic diseases in hospital and the wider community [12, 13].

Despite numerous initiatives, the documentation rate of ACDs has failed to significantly improve for three decades in Australia [6, 7, 14–16]. Given the concept retains strong support, a new approach was needed. Advance Care Planning (ACP) emerged as a process where individuals, family members and healthcare professionals discuss the individual’s values in life and goals for health care, and future treatment preferences for a time when they are not able to make health care decisions [17]. In New South Wales (NSW), Australia, there are two main outcomes from ACP; the written ACD and/or the appointment of an Enduring Guardian (EG) or identification of the Person Responsible (PR) as a substitute decision maker (SDM). It is ideal if ACDs are documented as a result of ACP when the person is still well and capable of making decisions [17].

A growing body of international literature concurs that comprehensive and complex ACP programs involving specially qualified or trained healthcare professionals, as facilitators, for example, social workers in Canada [18], General Practitioners in Australia [19], Registered Nurses (RNs) in the Netherlands [20] and Australia [21], are effective in increasing documentation of ACDs. Other studies reported that ACP improved compliance with patients’ wishes and satisfaction with care [6, 22, 23], and quality of care for patients and their families [24, 25].

However, one inconclusive aspect of ACDs and ACP is the impact of ACDs and ACP on the costs of care and economic benefits. Earlier studies that heavily focused on health care costs and resources are criticised for low documentation rates of ACDs. From the literature in the 1990s it was clear that the focus of studies was on the cost effectiveness of ACDs. Weeks et al. [26] found that the preferences of patients with ACDs are to limit care and that patients without ACDs have significantly higher terminal hospital charges than those with ACDs. In 2002, Taylor and Cameron [27] identified potential savings in medical resource expenditure via reductions in inpatient and outpatient acute hospital services; acute hospital care; therapeutic and ancillary services; and general medical practice. Even when the purpose of the ACP is not cost containment, there may be a philosophical emphasis at a policy level and/or within clinical decision-making processes on limiting, rather than maintaining treatment. For example, Gillick [28] described ACD as a method to avoid excessive and undesired interventions in the final years of life.

Recently, two systematic reviews [29, 30] on the economic aspects of ACP were conducted. The gaps identified in these reviews indicate the lack of evidence on the costs involved in ACP facilitation and interventions particularly for people who retain capacity, and the lack of methodologically robust trials with clearly defined ACP interventions. More recently, in Australia, Nguyen et al. [31] modelled the potential cost-effectiveness of an ACP intervention in a cohort of older people (aged 65+ years) who were at risk of cognitive decline. They determined that an intervention to encourage ACP completion would be cost saving, if completion rates were 50% or higher and adherence to ACP wishes were above 75%. This economic modelling demonstrated further the requirement for good evidence regarding the costs required to deliver ACD completion rates. Another gap identified in the systematic reviews [29, 30] includes the lack of studies conducted both in healthcare settings other than the United States, and in non-hospital settings.

### Aim

The aim of this analysis was to add to the evidence on resource use associated with implementation of ACP and to inform key decision-makers of the resource implications, through the conduct of a cost-consequence analysis of the normalised ACP (NACP) trial. The analysis builds upon a quasi-experimental study conducted in 2018 to investigate the effect, measured by a completed ACP document, of the NACP service intervention compared to usual care. The study protocol is reported elsewhere [4]. In this paper, we report the cost-consequence analysis of the NACP intervention compared to usual care.

## Methods

### Target population and setting

The target population comprised patients 1) with chronic diseases (defined within this research project as Cancer, Chronic Kidney Disease, Chronic Obstructive Pulmonary Disease, Congestive Heart Failure, Coronary Artery Disease, Dementia, Diabetes, Frailty and Hypertension), 2) aged ≥18 years old, admitted to the wards/community services in participating hospitals and community settings, and 3) without an existing ACD.

The trial was conducted in two care provision domains (the community care setting and the hospital setting) in two Local Health Districts (LHDs) within two metropolitan areas. The population covered by LHD 1 and LHD 2 was 920,370 and 339,550 people respectively. The settings contained a total of eight sites, which included two Geriatric Rehabilitation units, six Medical wards, four public Community Health Centres and four Non-Government Community Health services. Each LHD contained four intervention and four control sites which were pair-matched based on patient profile, admission rates, average length of stay, and number of deaths per year. The selection of interventions sites and the matched control sites is detailed in the trial protocol [4].

### The intervention

Prior to commencement of the intervention, all staff (e.g. Medical Officers, Registered Nurses (RNs), and Social Workers, if applicable) in the participating sites were invited to attend a 20–30 min information session about the research project. Four ACP RNs were recruited and completed a five-day training program. The training was a blend of online and face-to-face learning which included readings, reflections, role play and scenario-based case discussions. The intervention, (an ACP service), was offered by these RNs as part of routine/normalised service to all clients who were admitted to participating intervention sites. In hospital settings, on admission, potential participants were informed by the ACP RNs on site that “A new Advance Care Planning service is currently being offered to all new clients and their family. Are you interested in?”. In community settings, on admission, the admitting community RNs offered the new ACP service and those patients who expressed interest were referred to the ACP RNs for a home visit. The ACP RNs facilitated a series of conversations with those who accepted the ACP service about the components of ACP for the individual, their nominated Substitute Decision-Makers and other treating healthcare professionals (e.g. General Practitioners, Medical Officers) as required. A summary of outcomes of these conversations were entered: 1) in the person’s medical record (hard copy &/or online), and 2) on a ‘Conversation Card’ (A4 double-sided and a size of business card when folded) for carrying in wallet. The detailed description of the intervention is reported elsewhere [4].

### Ethics

This project has been approved by the Hunter New England Human Research Ethics Committee (Approval No. 17/12/13/4.16). Informed consent was sought and obtained for uptake of NACP service, and voluntary participation was ensured. The study was conducted in accordance with the National Health and Medical Research Council’s National Statement on Ethical Conduct in Human Research (2007), and under the governance of the Human Research Ethics Committee (HREC) at the University of Newcastle and the two Local Health Districts.

### Study perspective

The health economic analysis was conducted from the perspective of a health care provider. The use of a health care provider, as opposed to a health service, provides for the inclusion of non-government home and community care providers, to whom the service provision has been outsourced.

### Study boundary

It is assumed that the policy to justify the wider implementation of ACDs is founded on prior evidence of improved alignment with patient preferences. As a consequence, this analysis is confined to the resource implications associated with an implementation intervention seeking to realise completion of additional ACDs. As such, the study boundary excludes the requirement to account for downstream health service costs and/or health outcome considerations. The project management schedule and available research budget also necessitated imposition of this constraint.

### Comparators

The comparators for the economic analysis are consistent with the main trial. The comparators vary with the setting as described in Table 1.

**Table 1:** The description of Usual Practice and Intervention

For the community setting	For the hospital setting
Usual Practice: Community Registered Nurses (RNs) visit patients to provide initial needs assessment, wound care, injections, and other clinical services based on the needs identified. Patients may be given ACP information, but the dissemination is ad hoc, and a prior audit found minimal (0.4%) conduct of ACP and completion of ACDs [in review].	Usual Practice: Existing policy recommends that hospital RNs should introduce ACP to relevant inpatients. In reality, a brochure may be available within the department, but specific introduction of ACP to relevant patients is rarely (1.8%) conducted [in review].
Intervention: Two community ACP Registered Nurses (ACP RNs) were allocated, one per LHD catchment area. Each ACP RN was trained specifically in delivering of NACP service and documentation/completion of ACDs. • Step 1: Usual community RNs visited patients at their home for usual care. Community RNs applied inclusion/exclusion criteria for all new admissions. For eligible patients, the community RN introduced the one-page double-sided ACP brochure and asked if patients would like to use the free ACP service. If patients accept, the community RN gains formal consent and refers the patients to the community ACP RN. • Step 2: Community ACP RN contacted patients to arrange visits. Community ACP RNs visited patients (and potentially their carers) at home. On average one to three visits were conducted until an ACD was either declined or completed.	Intervention: Two ACP RNs were allocated to cover two wards in two public hospitals, one per LHD. Each ACP RN was trained specifically in delivering of NACP service and documentation/completion of ACDs. • Step 1: One-page double-sided ACP brochures were included in hospital admission documentation/information packs and were provided to all new admissions. • Step 2: ACP RNs reviewed patient journey boards each day on the wards and used inclusion/exclusion criteria to identify eligible patients from all new admissions. ACP RNs visited eligible inpatients and asked if patients would like to use the free ACP service.
• Step 3: Conversation process: ACP RNs ➢ initiated with open ended questions exploring the person’s knowledge, attitude and desire to participate in ACP ➢ identified who should be involved in conversations ➢ identified the person’s understanding of diagnosis, prognosis and preferences for treatment options and place of care ➢ facilitated a series of conversations between the person, the nominated SDM, treating medical team according to the responses above ➢ discussed and supported, where relevant, completion of ACDs ➢ captured the summary of conversations in Conversation Card.	

### Time horizon, discount rates and price data

The economic analysis represents a within-trial study. Consequently, the study time horizon accounts for the trial impacts up to the end-point and, as already noted, does not account for longer term health outcomes or health service utilisation. The short time period for both the trial and the analytical timeframe precluded the requirement to apply discount rates. All costs are reported in Australian dollar 2019 rates.

### Outcome measures

The outcomes for the economic evaluation include:

- the number of completed NSW ACDs; and
- the number of completed Conversation Cards (CC)

### Resource costs

The resource utilisation associated with the intervention was estimated retrospectively. Estimates were derived through the available project records/documentation. The analysis assessed the incremental difference in resource utilisation between Usual Practice and the Intervention. Costs have been categorised into; 1) Contract staff costs; 2) Costs associated with the development of the intervention; 3) Implementation costs; 4) Intervention (delivery) costs; and 5) Research costs.

1) Contract staff costs: The project contracted four staff members (Registered Nurses - RNA, RNB, RNC and RND) to deliver the intervention. Micro-costing data was unavailable to estimate the respective volume of resource use per task. Contract staff costs were treated as a lump sum to deliver specified tasks. The tasks conducted by these staff members were specified, within the implementation, intervention and research requirements. The allocated tasks were excluded from the other categories to avoid any double counting of expenditure. For example, intervention tasks conducted by the contract staff were explicitly excluded from the intervention (delivery) costs.

2) Intervention (development) costs: These costs were excluded from the main intervention costs because generalisation of the intervention, in its existing form, would probably not necessitate the recurrence of this expenditure. Inclusion of these costs in the main intervention costs would over-estimate the resource requirement.

3) Implementation costs: All implementation costs are additional (incremental) to Usual Practice. They include the costs incurred to train, educate, facilitate and enabled the implementation of the intervention. Examples include the training of ACP RNs and steps to provide access to health service databases.

4) Intervention (delivery) costs: Following detailing of Usual Practice and the Intervention, intervention (delivery) costs reflect the incremental difference in the resources required between the two comparators aggregated for the study population. Six patients in hospital in LHD 2 were referred to the ACP RN in community setting on discharge. When followed up, five patients declined the ACP service at home and only one patient completed an ACD with the ACP RN, which was included in the analysis. No patients in hospital were referred to the ACP RN in the community setting in LHD 1.

5) Research costs: Costs associated with the conduct of research were explicitly excluded. The proportional attribution of resources to the intervention and the research respectively were obtained from the project records and through discussions with the research team. For example, 95% of the cost of the mobile phone used by the community ACP RNs was attributed to the intervention and 5% to the conduct of the research.

On-costs, reflecting fringe benefits, and leave conditions were applied to all labour resources. ‘On-costs’ were preferred over ‘overheads’ to avoid double-counting with the costs explicitly identified in the analysis e.g. office space, IT infrastructure.

### Analytical method and structural assumptions

The economic evaluation comprised a simple cost-consequence analysis. The aggregate incremental additional cost was divided by the generated outcomes to estimate a cost per outcome. The analysis was also conducted following stratification by setting. Analysis of the costs was conducted and the evaluation reported according to best practice [32, 33]. The analysis is designed to produce evidence relevant to broader cost-effectiveness analysis of relevant programs.

## Results

### Study parameters

A number of parameter assumptions underpin the results. These generic assumptions are listed in Additional File 1. The costs incurred, due to refinement and development of the intervention development, are detailed in Additional File 2. The cost of the ACP RNs contracted to deliver the intervention are summarised in Additional File 3. The tasks and resources required to implement the intervention across both settings are detailed in Additional File 4. Details regarding the volume, value and component costs of the tasks and equipment required to deliver the intervention, including cross references to the tasks conducted by the contract staff, are detailed in Additional File 5. All labour costs have been increased by 17% to account for on-costs [34].

### Outcomes

The outcomes (the number of completed ACDs and Conversation Cards) from the trial are reported in Tables 2 and 3. The detailed statistical analyses of the outcomes between pre- and post-intervention and intervention vs control sites are provided in the main trial results [35]. In summary, for the economic analysis:

- The number of completed ACDs were higher (statistically significant, as per specified statistical analysis method) for the intervention. However, stratification of the analysis found that the intervention was only successful within the community setting.
- Conversation Cards (CC) were introduced as a novel component of the intervention. Consequently, the number of completed CCs in the intervention settings were all additional to the control arm. Stratification of the analysis demonstrates that the intervention was markedly more successful in the community setting, realising 89% of the total completed CCs. Please refer to the trial analysis for further information [35].

**Table 2:** Outcomes - Completed ACD by setting and trial arm

Setting	Trial arm	Outcomes (ACD): Pre	Outcomes (ACD): Post	Incremental Change
Community	Control	1	1	0
Community	Intervention	1	78	+ 77
Hospital	Control	4	1	−3
Hospital	Intervention	4	7	+ 3

**Table 3:** Outcomes – Number of complete Conversation Cards by setting and trial arm

Setting	Trial arm	Outcomes (Conv. Cards): Pre	Outcomes (Conv. Cards): Post	Incremental Change
Hospital	Control	0	0	0
Hospital	Intervention	0	13	+ 13
Community	Control	0	0	0
Community	Intervention	0	107	+ 107
Total	Control	0	0	0
Total	Intervention	0	120	+ 120

### Resource costs

The identified economic costs necessary for the refinement of the intervention included the development of training and education materials, and the development of materials to facilitate the discussion. The total estimate for the cost of intervention development was $3561 (Additional File 2). If the intervention was generalised in its current form, these costs may not be incurred in additional settings. To reflect this consideration, intervention development costs were excluded from the incremental cost analysis.

Table 4 Summarises the outcome measures, the aggregate of the staff contract, implementation and intervention (delivery) costs, and the resulting incremental cost per outcome

Total economic costs to deliver the specified intervention, excluding intervention development costs, measured $195,178 (Table 4). The cost of contract staff, at $171,448 (Additional File 3), comprised the largest component of the total costs. The estimated total resource use attributed to the community and the hospital strata was $97,317 and $97,861 respectively (Table 4). All labour costs, including contract staff, comprised 81% of the total. Out-sourced expenditure on goods or services (e.g. mobile phones) comprised 10% and the implicit provision of office space, transport and IT infrastructure comprised 9% of total economic costs respectively (Additional File 5).

**Table 4:** Incremental cost per outcome

	Additional Costs				Additional Outcomes		Incremental cost per outcome	
Stratification	Contracted	Implementation	Intervention (delivery)	Total	Conversation Cards	ACDs	Conversation Cards	Completed ACDs
All	$170,328	$5297	$19,552	$195,178	120	85	$1626	$2296
Community setting	$82,847	$2482	$11,987	$97,317	107	78	$910	$1248
Hospital setting	$87,481	$2815	$7565	$97,861	13	7	$7528	$13,980

The estimate for total implementation cost for the specified intervention was $5297 (Table 4). The majority of the costs were associated with training and the conduct of information sessions. These costs would need to be incurred if the intervention was generalised to any new settings and consequently, were included in the incremental cost analysis. It is probable that many components would also be required to sustain the intervention within the trial settings, given for example staff turnover or evolving service practice.

The value of the resources required to deliver the specified intervention, excluding the components delivered by the contracted staff and the implementation costs, was estimated at $19,552 (Table 4). The largest cost components comprised provision for office space, access to IT infrastructure and transportation. The estimated resource use attributed to the community and the hospital strata was $11,987 and $7565 respectively (Table 4). Intervention delivery within the community setting generated higher costs predominantly due to the transport required to attend meetings in the patient’s home ($4308) (Additional File 5).

### Cost-consequence analysis

This economic analysis is prefaced on the assumption that policy encouraging the application of ACP is founded on prior health outcome or resource cost evidence. Consequently, the analysis explicitly excluded the downstream health service impacts that may or may not arise from the realisation of additional ACDs. The incremental cost-consequence analysis (Table 4) reflects process metrics in the line with the research objective to realise additional completed CCs and/or additional ACDs.

In summary,

- Completed Conversation Cards

o $1626 per completed Conversation Cards (*n* = 120) across both settings.

o $910 per completed Conversation Cards (*n* = 107) within the community setting.

o $7528 per completed Conversation Cards (*n* = 13) within the hospital setting.

Note: The resource costs could not be disaggregated explicitly for the steps to deliver just completed Conversation Cards. Consequently, the estimated incremental cost per Conversation Cards incorporates the subsequent steps to realise ACDs, where pursued. This implies that the incremental cost per completed Conversation Cards should only be considered in context of the estimated incremental costs per ACD.

- Completed ACDs

o $2296 per completed ACD (*n* = 85) across both settings.

o $1248 per completed ACD (*n* = 78) within the community setting.

o $13,980 per completed ACD (n = 7) within the hospital setting.

## Discussion

Since the concept of ACDs was first proposed by Kutner in 1967 [36], and considering its evolution to ACP as a fully informed decision-making process for and towards end of life, a plethora of evidence reported the actual and potential benefits of ACPs. However, the evidence base regarding the economic merits of ACP/ACD has been controversial and inconclusive for over 30 years. O’Hanlon et al. [37] recommended that a comprehensive economic evaluation of ACP should consider implementation and intervention costs during the study period.

The cost-consequence analysis of this quasi-experimental trial adds new knowledge to the costs involved to implement an NACP service in hospital and community settings for people who retained capacity. This trial analysis found no evidence to infer that the specified intervention was effective in generating additional ACDs within hospital settings given that the number of ACDs completed in hospital settings only increased from one to four in LHD1 and was unchanged at three in LHD2. In contrast to the hospital setting, the number of ACDs completed in the community setting increased from 0 to 26 in LHD1 and from one to 52 in LHD2. Accordingly, the estimated mean average incremental cost per completed ACD in hospital settings is unlikely to be sustainable at $13,980. The reasons for the low uptake of NACP service in hospital settings are reported elsewhere [35]. Significant resources were incurred delivering the intervention within this setting. On this basis, this intervention should not be generalised within this, or similar hospital settings, without significant amendment or understanding regarding the basis for the failure to realise the anticipated outcomes.

The estimated mean average incremental cost per completed ACD for the community setting was $1248. In the absence of a decision rule, this analysis cannot recommend whether this estimate is favourable or otherwise. Two recent publications present alternative estimates for the delivery of ACP, and the realisation of completed ACDs, within broader cost-effectiveness analyses that account for health outcomes and downstream health services costs within an Australian context. Sellars et al. [38] conducted an analysis of an ACP initiative for older persons with end-stage kidney disease. They estimated the mean cost of ACP (as specified) at $519 per patient (price date unspecified, presumed to be in 2018 Australian dollars). Their modelling found that the cost of the ACP intervention made little difference to the incremental cost-effectiveness ratio, compared to other factors, such as the cost of care in the last 12 months of life, the probability of dying and the probability of adherence to treatment preferences. Their results found that ACP realized greater adherence to patient’s preference, but at a higher cost.

On the other hand, the ex-ante economic modelling conducted by Nguyen et al. [31] tested a cost range of AU$670-AU$820 (AUD2015) per individual ACP (as specified). Their study hypothesized a nationwide ACP program delivered in a primary care setting for people aged 65+ who are at risk of dementia. In contrast to Sellars et al. [38], their analysis found that an ACP intervention would dominate Usual Care, that is would improve outcomes and save costs, within certain key threshold assumptions. These assumptions include whether ACP completion was higher than 50%. The evidence from our NACP trial, regarding the percentage conversion between completed Conversation Cards and completed ACDs, infers that completion rates should be higher than this threshold for equivalent implementation interventions. Nguyen’s [31] modelling also found a threshold for the cost per ACP of $850 with compliance rate of 75% with end of life wishes, at which the ACP intervention in this study was no longer cost saving. Assuming all other assumptions were consistent, this would imply that the cost per completed ACD generated from the NACP trial, even in a community setting, may incur additional costs to generate the additional patient benefits regarding their preferred treatment pathway.

ACP has emerged as an important process for patients, families and health care professionals to work together to guide future care. However, this ideal goal is also in danger of being entangled with other issues, such as the pressure to reduce care at the end of life, and the economic pressures exerted by society and insurers to contain care costs. ACP as a means for economic rationalism rather than for optimising care of the individual is a legitimate concern for some, especially those with chronic conditions. However, the question of when and how treatments should be ceased can be clinically, socially and ethically complex. The focus of economic analysis here is to reflect the importance of individuals’ expressed personal preferences for care toward and at the end of life. The results from our analysis provide greater transparency for clinicians and decision makers to the costs involved in NACP implementation, and to make an assessment regarding the merits of using these resources to realise the incremental gains. It is recommended that this evidence regarding the incremental cost per ACD, be considered within the wider context of the subsequent health outcomes and downstream health service costs that may arise for people with or without an ACD. The extended models suggest that these considerations may be critical to determine the relative value of an ACD intervention compared to the alternative investments available to health service decision makers. An updated economic model that incorporates the evidence released since the prior analysis, including the results of this study, would appear valuable.

The increasing needs for health care services amid finite resources rightfully drive policy and practice for efficiency and effectiveness. It is important to remember that the objective of such economic evaluation is to optimise patient outcomes across the respective health budget, as opposed to inhibit service provision. This analysis adds new knowledge to the field regarding the potential cost thresholds for the realisation of completed ACDs and the relative merits of their implementation within hospital or community settings. Further experimental implementation studies, seeking to generalise ACP service, should expand the study boundary to account for implementation costs, intervention costs and the implications for health care service provision.

### Limitations

The limitations of this economic analysis include the retrospective approach, which relied upon interviews and administrative records of the project team to determine the resource categories and volume of resource use and proportional allocation to the intervention. Specifically, detailed records to micro-cost the resources required to conduct specific tasks undertaken by the ACP RNs (contract staff) were not available. The analysis consequently assumes that the tasks conducted by the ACP RNs, while specified, equate to the resources required to contract these roles, which may or may not be appropriate. In the absence of further detail, tasks relevant to both settings were attributed 50% to either setting, an assumption which was supported by the implementation team. The cluster design, particularly following stratification, provided no distribution ranges for outcomes from which to derive any meaningful sensitivity or uncertainty estimates. Similarly, the retrospective approach, the study boundary and the lack of granularity in the contract staff time constrains any meaningful examination of the results to changes in the cost assumptions.

## Conclusion

The economic analysis does not support generalisation of the specified intervention within the hospital setting. Although qualified by the study limitations, the trial realised an estimated incremental cost per completed ACD of $1264, within the community setting. This estimate provides an additional benchmark against which decision-makers can assess the value of either:

- this approach to the realisation of additional completed ACDs; and/or
- the value of ACP and ACDs more broadly, when this estimate is positioned within the potential health outcomes and downstream health service implications that may arise for people with or without a completed ACD.

## Supplementary Information

**Additional file 1.**

**Additional file 2.**

**Additional file 3.**

**Additional file 4.**

**Additional file 5.**

## Data Availability

All data relevant to the cost-consequence analysis for this study are analysed and included in this published article.

## Abbreviations

ACP: Advance Care Planning
ACD: Advance Care Directive
CC: Conversation Cards
EG: Enduring Guardian
PR: Person Responsible
RN: Registered Nurse
SDM: Substitute Decision Maker
