# Supplementary material for: A cost-consequence analysis of normalised advance care planning practices among people with chronic diseases in hospital and community settings
Source: BMC Health Serv Res. 2021 Jul 23;21:729. doi: 10.1186/s12913-021-06749-x (PMC8305493; doi:10.1186/s12913-021-06749-x)
Supplement: Supplementary file 1 — Additional file 1. [file 12913_2021_6749_MOESM1_ESM.docx]

Additional File 1. General assumptions – Staff hourly costs

| **Labour - Staff description** | **Cost per hour***  **(AUD2019)** | **Source** |
| --- | --- | --- |
| RNA: 5th year (Inpatient ACP RN LHD 1) | $38.69 | Public Health System Nurses' & Midwives' (State) Award 2018 1 July 2018 |
| RNB^a^: 2nd year (Community ACP RN LHD 1) | $33.30 | Public Health System Nurses' & Midwives' (State) Award 2018 1 July 2018 |
| RNC: 8 years plus (Inpatient ACP RN LHD 2) | $44.34 | Public Health System Nurses' & Midwives' (State) Award 2018 1 July 2018 |
| RND: 8 years plus (Community ACP RN LHD 2) | $44.34 | Public Health System Nurses' & Midwives' (State) Award 2018 1 July 2018 |
| RNs (unspecified - assumed 4th year average) | $36.86 | Public Health System Nurses' & Midwives' (State) Award 2018 1 July 2018 |
| Chief Investigator (CI) 1: NM Level 3 | $59.84 | Public Health System Nurses' & Midwives' (State) Award 2018 1 July 2018 |
| Chief Investigator (CI) 2: Academic | $74.51 | [Combined Academic Staff and Teachers Enterprise Agreement 2018 (Schedule 1 Part A)](https://www.newcastle.edu.au/__data/assets/pdf_file/0004/517180/Combined-Academic-Staff-and-Teachers-Enterprise-Agreement-2018.pdf%20(Schedule%201%20Part%20A)) |
| Associate Investigator (AI) 1: NM Level 3 | $59.84 | Public Health System Nurses' & Midwives' (State) Award 2018 1 July 2018 |
| Associate Investigator (AI)2: Staff specialist – Geriatrician | $103.98 | Staff Specialists (State) Award 3 July 2018 |
| Medical Officers (MO): Assumed average: Grade 1 Year 2 | $67.83 | Public Health System Nurses' & Midwives' (State) Award 2018 1 July 2018 |
| Enrolled Nurse (EN): 5th Year and Thereafter/Case coordinator role | $30.93 | Public Health System Nurses' & Midwives' (State) Award 2018 1 July 2018 |
| Associate Investigator (AI) 4 - Staff specialist | $103.98 | Staff Specialists (State) Award 3 July 2018 |
| Clinical Nurse Consultant: Grade 2 - 2nd Year >31.12.99 | $58.87 | Public Health System Nurses' & Midwives' (State) Award 2018 1 July 2018 |

* Excluding on-costs; ^a^The original plan was to recruit and train ACP RNs from the existing RNs as a secondment within the LHDs for sustainability of the ACP service. However, due to the shortage of RNs in both LHDs, four ACP RNs were recruited via an external competitive process. The community ACP RN in LHD 1 had 2.5 years of experience as an RN at the time of appointment but had previously worked as an Assistant In Nursing (a health care worker who supports the delivery of nursing care by assisting people with personal care and activities of daily living under the supervision of RN) for 12 years.
