# Supplementary material for: A cost-consequence analysis of normalised advance care planning practices among people with chronic diseases in hospital and community settings
Source: BMC Health Serv Res. 2021 Jul 23;21:729. doi: 10.1186/s12913-021-06749-x (PMC8305493; doi:10.1186/s12913-021-06749-x)
Supplement: Supplementary file 2 — Additional file 2. [file 12913_2021_6749_MOESM2_ESM.docx]

Additional File 2: Resource utilisation (Intervention development)

| **Description** | **Category** | **Sub-category** | **Resources** | **Intervention strata (Community / Inpatient / Both)** | **Cost per unit (AUD2019)** | **Unit Type** | **Volume per event** | **No. of events** | **Allocation to intervention (i.e. excl. research)** | **Sub-total cost*** | **Additional sources & assumptions** |
| --- | --- | --- | --- | --- | --- | --- | --- | --- | --- | --- | --- |
| Development of education materials for ACP RNs | Labour | RNA: 5th year | Included in RNA TOTAL | Both | $59.84 | Hours | 16.00 | 1 | 100% | $1,120.14 |  |
| Development of education materials for ACP RNs | Labour | CI2 | Time | Both | $74.51 | Hours | 16.00 | 1 | 100% | $1,394.90 |  |
| Scenario development (Culturally and linguistically diverse (CALD)) | Labour | CI2 | Time | Both | $74.51 | Hours | 4.00 | 1 | 100% | $348.73 |  |
| Training development - Grief, loss & relief, Gerotranscendence | Labour | CI2 | Time | Both | $74.51 | Hours | 4.00 | 1 | 100% | $348.73 |  |
| Intervention documents: Research team-developed Conversation Card | Labour | CI2 | Time | Both | $74.51 | Hours | 4.00 | 1 | 100% | $348.73 |  |
|  |  |  |  |  |  |  |  |  | Total | $3,561.23 |  |

*Notes: Includes on-costs
