# Supplementary material for: A cost-consequence analysis of normalised advance care planning practices among people with chronic diseases in hospital and community settings
Source: BMC Health Serv Res. 2021 Jul 23;21:729. doi: 10.1186/s12913-021-06749-x (PMC8305493; doi:10.1186/s12913-021-06749-x)
Supplement: Supplementary file 3 — Additional file 3. [file 12913_2021_6749_MOESM3_ESM.docx]

Additional File 3: Resource utilisation (Contracted staff)

| **Description** | **Category** | **Sub-category** | **Resources** | **Intervention strata (Community / Inpatient / Both)** | **Cost per unit**  **(AUD2019)** | **Unit Type** | **No. of events** | **Allocation to intervention (i.e. excl. research)** | **Sub-total cost*** |
| --- | --- | --- | --- | --- | --- | --- | --- | --- | --- |
| Inpatient ACP RN: LHD 1 | Labour | RNA: 5th year | Contracted staff | Inpatient | $1,470.20 | Weeks | 24 | 100% | $41,283.22 |
| Community ACP RN: LHD 2 | Labour | RNB: 2nd year | Contracted staff | Community | $1,265.30 | Weeks | 24 | 100% | $35,529.62 |
| Inpatient ACP RN: LHD 2 | Labour | RNC: 8 years plus | Contracted staff | Inpatient | $1,685.10 | Weeks | 24 | 100% | $47,317.61 |
| Community ACP RN: LHD 2 | Labour | RND: 8 years plus | Contracted staff | Community | $1,685.10 | Weeks | 24 | 100% | $47,317.61 |
|  |  |  |  |  |  |  |  | Total | $171,448.06 |

*Notes: Includes on-costs
