# Supplementary material for: A cost-consequence analysis of normalised advance care planning practices among people with chronic diseases in hospital and community settings
Source: BMC Health Serv Res. 2021 Jul 23;21:729. doi: 10.1186/s12913-021-06749-x (PMC8305493; doi:10.1186/s12913-021-06749-x)
Supplement: Supplementary file 4 — Additional file 4. [file 12913_2021_6749_MOESM4_ESM.docx]

Additional File 4: Resource utilisation (Implementation costs)

| **Description** | Category | Sub-category | Resources | Intervention strata (Community / Inpatient / Both) | Cost per unit (AUD2019) | Unit Type | Volume per event | No. of events | Allocation to intervention (i.e. excluding research) | Sub-total cost* | Additional sources & assumptions |
| --- | --- | --- | --- | --- | --- | --- | --- | --- | --- | --- | --- |
| Project Management - Corporate orientation; access to computers and datasets (CHIME, iPMS); Site orientation | Labour | Registered Nurse (RNA - RN Level 5) | Included in RNA TOTAL | Inpatient | n/a | n/a | n/a | n/a | 100% |  |  |
| Project Management - Corporate orientation; access to computers and datasets (CHIME, iPMS); Site orientation | Labour | Registered Nurse (RN 2 - RN Level 2) | Included in RNB TOTAL | Community | n/a | n/a | n/a | n/a | 100% |  |  |
| Project Management - Corporate orientation; access to computers and datasets (CHIME, iPMS); Site orientation | Labour | AI1 | Time | Both | $59.84 | Hours | 1.00 | 1 | 100% | $70.01 |  |
| Project Management - Corporate orientation; access to computers and datasets (EMR); Site orientation | Labour | Registered Nurse (RNC - RN Level 8+) | Included in RNC TOTAL | Inpatient | n/a | n/a | n/a | n/a | 100% |  |  |
| Project Management - Corporate orientation; access to computers and datasets (EMR); Site orientation | Labour | Registered Nurse (RNC - RN Level 8+) | Included in RND TOTAL | Community | n/a | n/a | n/a | n/a | 100% |  |  |
| Project Management - Corporate orientation; access to computers and datasets (EMR); Site orientation | Labour | CI1 - Nurse Manager (Level 3) |  | Both | $59.84 | Hours | 1.00 | 1 | 100% | $70.01 |  |
| Research Project Information session (Intro. Session, role, recruitment overview, planned data log - ACP service delivery, other data collection) | Labour | AI1 (Nurse Manager - Level 3) | Included in RNC TOTAL | Both | $59.84 | Hours | 1.50 | 1 | 100% | $70.01 |  |
| Research Project Information session (Intro. Session, role, recruitment overview, planned data log - ACP service delivery, other data collection) | Labour | CI2 | Included in RND TOTAL | Both | $74.51 | Hours | 5.00 | 1 | 100% | $87.18 | Preparation of presentation slides; etc (2 hrs), delivered to both LHD 1 & 2 |
| Research Project Information session (participation) | Labour | RNA | Included in RNA TOTAL | Inpatient | n/a | n/a | 1.50 | n/a | 100% | n/a |  |
| Research Project Information session (participation) | Labour | RNB | Included in RNB TOTAL | Community | n/a | n/a | 1.50 | n/a | 100% | n/a |  |
| Research Project Information session (participation) | Labour | RNC | Included in RNC TOTAL | Inpatient | n/a | n/a | 1.50 | n/a | 100% | n/a |  |
| Research Project Information session (participation) | Labour | RND | Included in RND TOTAL | Community | n/a | n/a | 1.50 | n/a | 100% | n/a |  |
| Training: Compulsory Online training & other resources | Labour | RNA | Included in RNA TOTAL | Inpatient | n/a | n/a | Not available | n/a | 100% | n/a | On-line training and provided with other appropriate reading material/guidance |
| Training: Compulsory Online training & other resources | Labour | RNB | Included in RNB TOTAL | Community | n/a | n/a | Not available | n/a | 100% | n/a | On-line training and provided with other appropriate reading material/guidance |
| Training: Compulsory Online training & other resources | Labour | RNC | Included in RNC TOTAL | Inpatient | n/a | n/a | Not available | n/a | 100% | n/a | On-line training and provided with other appropriate reading material/guidance |
| Training: Compulsory Online training & other resources | Labour | RND | Included in RND TOTAL | Community | n/a | n/a | Not available | n/a | 100% | n/a | On-line training and provided with other appropriate reading material/guidance |
| Information Session for MOs run by Geriatrician: n=1; Role for intervention | Labour | AI2 (Staff specialist – Geriatrician) | Time | Inpatient | $103.98 | Hours | 0.30 | 1 | 100% | $36.50 |  |
| Information Session with Medical Officers: n=18 attendees in one hospital; Role for intervention | Labour | Medical Officers (MO - Junior officer average) | Time | Inpatient | $67.83 | Hours | 0.30 | 18 | 100% | $428.55 |  |
| Information Session for RNs run by CI2: n=1; community settings; Role for intervention | Labour | CI2 | Time | Community | $74.51 | Hours | 0.30 | 1 | 100% | $26.15 |  |
| Information Session with RN: n=12 time; one community setting; Role for intervention | Labour | RNs (unspecified) | Time | Community | $36.86 | Hours | 0.30 | 12 | 100% | $155.25 |  |
| Information Session for MOs run by Staff specialist: n=1; Role for intervention | Labour | AI4 (Staff specialist) | Time | Inpatient | $103.98 | Hours | 0.30 | 1 | 100% | $36.50 |  |
| Information Session with Medical Officers: n=18 attendees in one hospital; Role for intervention | Labour | Medical Officers (MO - Junior officer average) | Time | Inpatient | $67.83 | Hours | 0.30 | 18 | 100% | $428.55 |  |
| Information Session for RNs run by CI2: n=1; community settings; Role for intervention | Labour | CI2 | Time | Community | $74.51 | Hours | 0.30 | 1 | 100% | $26.15 |  |
| Information Session with RN: n=15 time; one community setting; Role for intervention | Labour | RNs (unspecified) | Time | Community | $36.86 | Hours | 0.30 | 15 | 100% | $194.06 |  |
| Information Session with 3 Case coordinators (Not RNs-Home community provider; non-govt provider): n=1 times two (each setting) | Labour | AI1 | Time | Community | $59.84 | Hours | 1.50 | 1 | 100% | $105.01 | Includes travel & CC guidance |
| Information Session with 3 Case coordinators (Not RNs-Home community provider; non-govt provider): n=3 & 2 (respective settings) | Labour | EN | Time | Community | $30.93 | Hours | 0.50 | 5 | 100% | $90.47 | Source: salary AIN Certificate IV Health care providers |
| Human Resources: Recruitment of RN ACP facilitators; role documentation for line manager (secondment) | Labour | AI1 | Time | Both | $59.84 | Hours | 4.00 | 4 | 100% | $1,120.14 | Four staff for four hours |
| Training: Facilitation (preparation, venues, etc.) | Labour | AI1 | Time | Both | $59.84 | Hours | 1.00 | 1 | 100% | $70.01 |  |
| Human Resources: Coordination of office space, site visit, etc. | Labour | AI1 | Time | Both | $59.84 | Hours | 8.00 | 1 | 100% | $560.07 |  |
| Training: Role-play, Scenario-Based Learning with ACP experts | Labour | AI1 | Time | Both | $59.84 | Hours | 5.00 | 1 | 100% | $350.05 |  |
| Training: Role-play, Scenario-Based Learning with ACP experts | Labour | CI2 | Time | Both | $74.51 | Hours | 5.00 | 1 | 100% | $435.91 |  |
| Training: Topic experts - Scenario-Based Learning with ACP topic experts | Labour | CNC | Time | Both | $58.87 | Hours | 3.00 | 1 | 100% | $206.65 |  |
| Training: Topic experts - Scenario-Based Learning with ACP topic experts | Labour | Staff Specialist - Geriatrician | Time | Both | $103.98 | Hours | 3.00 | 2 | 100% | $729.91 |  |
|  |  |  |  |  |  |  |  |  | Total | $5,297.13 |  |

*Notes: Includes on-costs for labour; n/a – not applicable
