# Supplementary material for: A cost-consequence analysis of normalised advance care planning practices among people with chronic diseases in hospital and community settings
Source: BMC Health Serv Res. 2021 Jul 23;21:729. doi: 10.1186/s12913-021-06749-x (PMC8305493; doi:10.1186/s12913-021-06749-x)
Supplement: Supplementary file 5 — Additional file 5. [file 12913_2021_6749_MOESM5_ESM.docx]

Additional File 5: Resource utilisation (Intervention delivery – Incremental difference between ACP intervention and Usual Practice)

| **Description** | Category | Sub-category | Resources | Intervention strata (Community / Inpatient / Both) | Cost per unit | Unit Type | Volume per event | No. of events | Allocation to intervention (i.e. excluding research) | Sub-total cost* | Additional sources & assumptions |
| --- | --- | --- | --- | --- | --- | --- | --- | --- | --- | --- | --- |
| Project management: Weekly debriefing & trouble shooting | Labour | RNA | Included in RNA TOTAL | Inpatient | n/a | Hours | 1.00 | 20 | 100% | n/a |  |
| Project management: Weekly debriefing & trouble shooting | Labour | RNB | Included in RNB TOTAL | Community | n/a | Hours | 1.00 | 20 | 100% | n/a |  |
| Project management: Weekly debriefing & trouble shooting | Labour | RNC | Included in RNC TOTAL | Inpatient | n/a | Hours | 1.00 | 20 | 100% | n/a |  |
| Project management: Weekly debriefing & trouble shooting | Labour | RND | Included in RND TOTAL | Community | n/a | Hours | 1.00 | 20 | 100% | n/a |  |
| NACP Intervention (Inpatient): Conversations with patients, service documentation, telephone patients, photocopy | Labour | RNA | Included in RNA TOTAL | Inpatient | n/a | Weeks | 20.00 | 1 | 100% | n/a | Total = 24 weeks; two weeks orientation; two weeks closing period |
| NACP Intervention (community): Conversations with patients, service documentation, telephone patients, photocopy | Labour | RNB | Included in RNB TOTAL | Community | n/a | Weeks | 20.00 | 1 | 100% | n/a | Total = 24 weeks; two weeks orientation; two weeks closing period |
| NACP Intervention (Inpatient): Conversations with patients, service documentation, telephone patients, photocopy | Labour | RNC | Included in RNC TOTAL | Inpatient | n/a | Weeks | 20.00 | 1 | 100% | n/a | Total = 24 weeks; two weeks orientation; two weeks closing period |
| NACP Intervention (community): Conversations with patients, service documentation, telephone patients, photocopy | Labour | RND | Included in RND TOTAL | Community | n/a | Weeks | 20.00 | 1 | 100% | n/a | Total = 24 weeks; two weeks orientation; two weeks closing period |
| Project administration: Troubleshooting, site visit, reflection, debriefing | Labour | Assoc. Professor Level D |  | Both | $74.51 | Hours | 20.00 | 1 | 100% | $1,743.63 |  |
| Intervention documents: NSW Health Advanced Care Directive booklets | Implicit | Cost - Advance Care Directive booklets | A4, 20-page, colour, double-sided; card cover | Both | $3.60 | per booklet | 1.00 | 150 | 100% | $540.00 | Provided by NSW Health, but costs estimated (https://www.print2day.com.au/stapled-booklets/a4-booklets-with-card-cover.html; excl GST); Development costs excluded |
| Intervention documents: Advanced Care Plan Flyer | Out-sourced | Production and printing | A4, one page, colour, double-sided; 1,400 copies | Both | $343.00 | per total order | 1.00 | 1 | 100% | $343.00 | Costs estimated (https://www.print2day.com.au/stapled-booklets/a4-booklets-with-card-cover.html; excl GST); Development costs excluded |
| Intervention documents: Research team-developed Conversation Card | Out-sourced | Production and printing | A4, one page, colour, double-sided; 200 copies | Both | $135.00 | per total order | 1.00 | 1 | 100% | $135.00 | Costs estimated (https://www.print2day.com.au/stapled-booklets/a4-booklets-with-card-cover.html; excl GST); (accessed 1 Dec 2019); Development costs excluded |
| Mobile phone for community ACP RNs | Out-sourced | Mobile phone contract | Basic phone and calls | Community | $60.00 | per mobile phone | 1.00 | 2 | 95% | $114.00 |  |
| Transport (car): In-kind support from the Community Health Centre (CHC) | Implicit | Transport to/from patient appointments | Car usage for five months (40% usage) | Community | $1,077.00 | per month | 5.00 | 2 | 40% | $4,308.00 | [http://www.mylease.com.au/short-term-rentals.aspx (1 Dec 2019); Toyota Corella (incl insurance, etc.; excl petrol)](http://www.mylease.com.au/short-term-rentals.aspx%20(1%20Dec%202019);%20Toyota%20Corella%20(incl%20insurance,%20etc.;%20excl%20petrol)); long term lease  (accessed 1 Dec 2019) |
| Office space: In-kind support from the Community Health Centres (CHC) | Implicit | Shared desk | Equivalent - Five months lease | Community | $517.00 | per desk space per month | 5.00 | 2 | 95% | $4,911.50 | <https://www.dashworks.com.au/workspace/>; one person (accessed 1 Dec 2019) |
| Office space: In-kind support from the Hospitals | Implicit | Shared desk | Equivalent - Five months lease | Inpatient | $517.00 | per desk space per month | 5.00 | 2 | 95% | $4,911.50 | [Partial research allocation; https://www.dashworks.com.au/workspace/; one person](file:///\\data.hmri.com.au\HMRI\Research%20Economists\HRE%20Projects\2019\CCLHD_SeOkOhr_TRGS_AdvCarePlanng\Deliverables\Reports\Partial%20research%20allocation;%20https:\www.dashworks.com.au\workspace\;%20SO%20TO%20PROVIDE) (accessed 1 Dec 2019) |
| Computer/other stationary: In-kind support from the Community Health Centre (CHC) | Implicit | Shared computer (stationary assumed negligible) | Equivalent - Five months lease | Community | $2.89 | per desk. pc per day | 440.00 | 2 | 50% | $1,272.92 | [https://h20386.www2.hp.com/AustraliaStore/Merch/Offer.aspx?p=leasing (accessed 1 Dec 2019)](https://h20386.www2.hp.com/AustraliaStore/Merch/Offer.aspx?p=leasing%20(accessed%201%20Dec%202019)) |
| Computer/other stationary: In-kind support from the Hospital | Implicit | Shared computer (stationary assumed negligible) | Equivalent - Five months lease | Inpatient | $2.89 | per desk. pc per day | 440.00 | 2 | 50% | $1,272.92 | https://h20386.www2.hp.com/AustraliaStore/Merch/Offer.aspx?p=leasing (accessed 1 Dec 2019) |
|  |  |  |  |  |  |  |  |  | Total | $19,552.47 |  |

*Notes: Includes on-costs for labour; n/a – not applicable
